# Clinical Audit of Enteric Fever Diagnostic Practices at Kosti Teaching Hospital: A Retrospective Evaluation

**DOI:** 10.7759/cureus.102301

**Published:** 2026-01-26

**Authors:** Ziryab Imad, Mohammed Fatahalla Khalid Malik, Marwa Mohammed Alfadel Kalol, Jamela Elsiddig Ali Elsiddig, Amna Yousif Mohammed Yassin, Ahmed S Yoseif, Babiker Omer Babiker Ibrahim, Asmaa Abdallah Mohammad Saeed, Enas Mohammed Ahmed Didi, Marwa Mohammed Almustfa Ibrahim, Esra Ebrahim Abdalhakam Fadalalmoula, Mustafa Awad, Tarig Alhadi Madibbo Ahmed

**Affiliations:** 1 Internal Medicine, Haj Al-Safi Teaching Hospital, Khartoum North, SDN; 2 Internal Medicine, University of Bahri, Khartoum North, SDN; 3 Medicine and Surgery, Faculty of Medicine, University of El Imam El Mahdi, Kosti, SDN; 4 Internal Medicine, Atbara Teaching Hospital, Atbara, SDN; 5 General Practice, Kosti Teaching Hospital, Kosti, SDN; 6 Orthopedics and Traumatology, Omdurman Teaching Hospital, Omdurman, SDN; 7 General Medicine, Kosti Teaching Hospital, Kosti, SDN; 8 General Practice, Omdurman Teaching Hospital, Omdurman, SDN; 9 Internal Medicine, Sudan Medical Specialization Board, Khartoum, SDN; 10 Medicine, Ziryab Research Group, Khartoum, SDN

**Keywords:** clinical audit, infectious diseases, internal medicine, quality improvement process, typhoid fever

## Abstract

Background and objective

Enteric fever remains a major public health problem in Sudan, particularly in areas with poor sanitation and limited health care resources. Accurate diagnosis is essential for appropriate management; however, diagnostic practices often rely on unreliable methods. This clinical audit aimed to evaluate current diagnostic practices for enteric fever at Kosti Teaching Hospital, assess adherence to WHO standards, identify gaps in diagnostic methods, and propose recommendations to improve diagnostic accuracy.

Methods

This study was a retrospective clinical audit conducted at Kosti Teaching Hospital, Sudan, reviewing the medical records of patients diagnosed with enteric (typhoid) fever during the study period. Data were extracted using a structured audit checklist to evaluate diagnostic practices, including documentation of clinical features, laboratory investigations requested (e.g., Widal/Felix-Widal test, serology, antibody testing, blood culture, and stool or rectal swab culture), and adherence to recommended diagnostic guidelines. Descriptive analysis was performed, and results were summarized using frequencies and percentages.

Results

Fifty-six patient records were reviewed. Headache and body pain (87.5%) and fever (85.7%) were the most common presenting symptoms. The Widal test was the most frequently used diagnostic tool (67.9%), followed by antibody detection (22.6%) and serology (Widal test) (17%). Blood culture, the diagnostic gold standard, was performed in only 1.9% of cases. No patients underwent stool culture or typhoid testing. The heavy reliance on the Widal test and clinical features, with minimal use of confirmatory tests, indicates substantial deviation from international guidelines.

Conclusions

The audit revealed significant gaps in enteric fever diagnosis at Kosti Teaching Hospital, characterized by heavy reliance on clinical assessment and Widal testing, with minimal use of blood culture due to limited resources. These practices compromise diagnostic accuracy and reflect broader health system challenges, including inadequate laboratory capacity and underfunding. Strengthening diagnostic infrastructure, improving access to reliable tests, and providing ongoing health care worker training are essential to align practices with WHO standards and improve patient outcomes in Sudan.

## Introduction

Enteric fever, or typhoid fever, is a systemic infection caused by *Salmonella enterica *serovars Typhi and Paratyphi. Transmission occurs mainly through ingestion of contaminated water or food, making it a persistent public health concern in low- and middle-income countries with poor sanitation and limited access to clean water [1,2]. Globally, the disease accounts for an estimated 9.9 million cases and 110,000 deaths annually, with the greatest burden in South Asia and Sub-Saharan Africa, where surveillance systems often underestimate its true impact [3-6].

In Sudan, enteric fever remains endemic, exacerbated by recurrent flooding, fragile sanitation infrastructure, and constrained healthcare resources [7,8]. Kosti Teaching Hospital, located in White Nile State, serves as a referral center for populations at high risk of infectious diseases, including typhoid [9,10]. Despite the significance of the disease, diagnosis in this setting largely relies on syndromic approaches rather than laboratory confirmation, predisposing patients to misdiagnosis and inappropriate treatment [11,12].

Accurate diagnosis is crucial for effective management; however, several barriers persist. These include limited diagnostic infrastructure, undertrained healthcare workers, and inconsistent application of guidelines [13,14]. Clinical audits provide a structured means of evaluating diagnostic practices against evidence-based standards and identifying opportunities for improvement [15,16]. The present audit at Kosti Teaching Hospital, therefore, aimed to examine diagnostic approaches, compare them with WHO guidelines, and provide actionable recommendations [17,18].

Pathophysiologically, *Salmonella Typhi *and *Salmonella Paratyphi *are human-adapted pathogens transmitted via the fecal-oral route [19,20]. After ingestion, the bacteria penetrate the intestinal mucosa, spread via the lymphatic system, and establish systemic infection, leading to high fever, abdominal pain, gastrointestinal symptoms, hepatosplenomegaly, and potentially severe complications such as intestinal perforation and septicemia [21,22].

Laboratory confirmation typically requires isolation of the organism from blood, bone marrow, or other sterile body fluids. Blood culture is regarded as the diagnostic gold standard, offering near-perfect specificity but limited sensitivity (40-60%), particularly if antibiotics are administered prior to sampling [23-25]. Alternative tests, including Widal serology and rapid diagnostic tests (RDTs), are frequently used in low- and middle-income countries (LMICs) but have variable accuracy [26,27].

In Sudan, systemic challenges further complicate diagnosis. A Khartoum-based study found that 60% of typhoid cases were diagnosed clinically without confirmatory testing [28]. Such reliance increases the risk of misdiagnosis and overuse of antibiotics, fueling the emergence of multidrug-resistant (MDR) *S. Typhi *strains [29,30]. At Kosti Teaching Hospital, diagnostic protocols remain inconsistently applied, and laboratories face resource limitations, reflecting a broader national problem.

Experiences from similar resource-constrained settings suggest that implementing standard operating procedures (SOPs), strengthening laboratory capacity, and training healthcare providers can substantially improve diagnostic accuracy and patient outcomes [31]. This audit, therefore, aims not only to assess current practices but also to provide insights that could inform policy and practice, both locally and in comparable LMIC contexts.

Enteric fever remains a pressing public health issue in Sudan. The combination of inadequate infrastructure, limited diagnostic tools, and overreliance on syndromic diagnosis undermines effective management. Conducting a clinical audit at Kosti Teaching Hospital is a critical step toward bridging the gap between current practices and WHO standards, ultimately improving patient outcomes and reducing the burden of typhoid in the region.

## Materials and methods

Audit design and setting

A retrospective clinical audit was conducted to evaluate diagnostic practices for enteric fever at Kosti Teaching Hospital, a major healthcare facility in White Nile State, Sudan, serving a population with a high burden of enteric fever.

Audit population and eligibility criteria

Medical records of patients diagnosed with enteric fever between January 2023 and January 2025 were reviewed. Patients of all age groups were included regardless of other medical conditions. Records were excluded if they contained incomplete or missing essential data, such as absent diagnosis, laboratory results, or treatment documentation, or if the diagnosis was uncertain or later revised to a non-enteric fever condition. After applying these criteria, the final sample consisted of 56 patient records.

Audit standard and data collection

Diagnostic criteria were based on the WHO Typhoid and Paratyphoid fact sheet, which specifies clinical symptoms and laboratory investigations for typhoid fever. Symptoms considered included low- to high-grade fever, headache and body pain, loss of appetite and weight loss, dry cough, sweating, abdominal pain, abdominal distention, diarrhea or constipation, and itching or rashes. Laboratory investigations included blood culture, serology, stool or rectal swab culture, the Felix-Widal test, and the Typhidot^®^ test [17].

A structured checklist was developed based on these criteria to extract data from patient records. Collected information included demographic details, clinical presentation, and diagnostic methods employed, both clinical and laboratory.

Data management and analysis

Data were cleaned and analyzed using Microsoft Excel 2016 (Microsoft Corporation, Redmond, WA, USA). Descriptive statistics summarized demographic and clinical data. Compliance with WHO diagnostic standards was assessed, and trends in antibiotic use were analyzed. Comparisons between diagnostic outcomes and laboratory-confirmed cases were performed to identify discrepancies.

Ethical considerations

Ethical approval was obtained from the institutional review board of Kosti Teaching Hospital. Patient confidentiality was maintained, and all data were anonymized.

## Results

**Figure 1:**
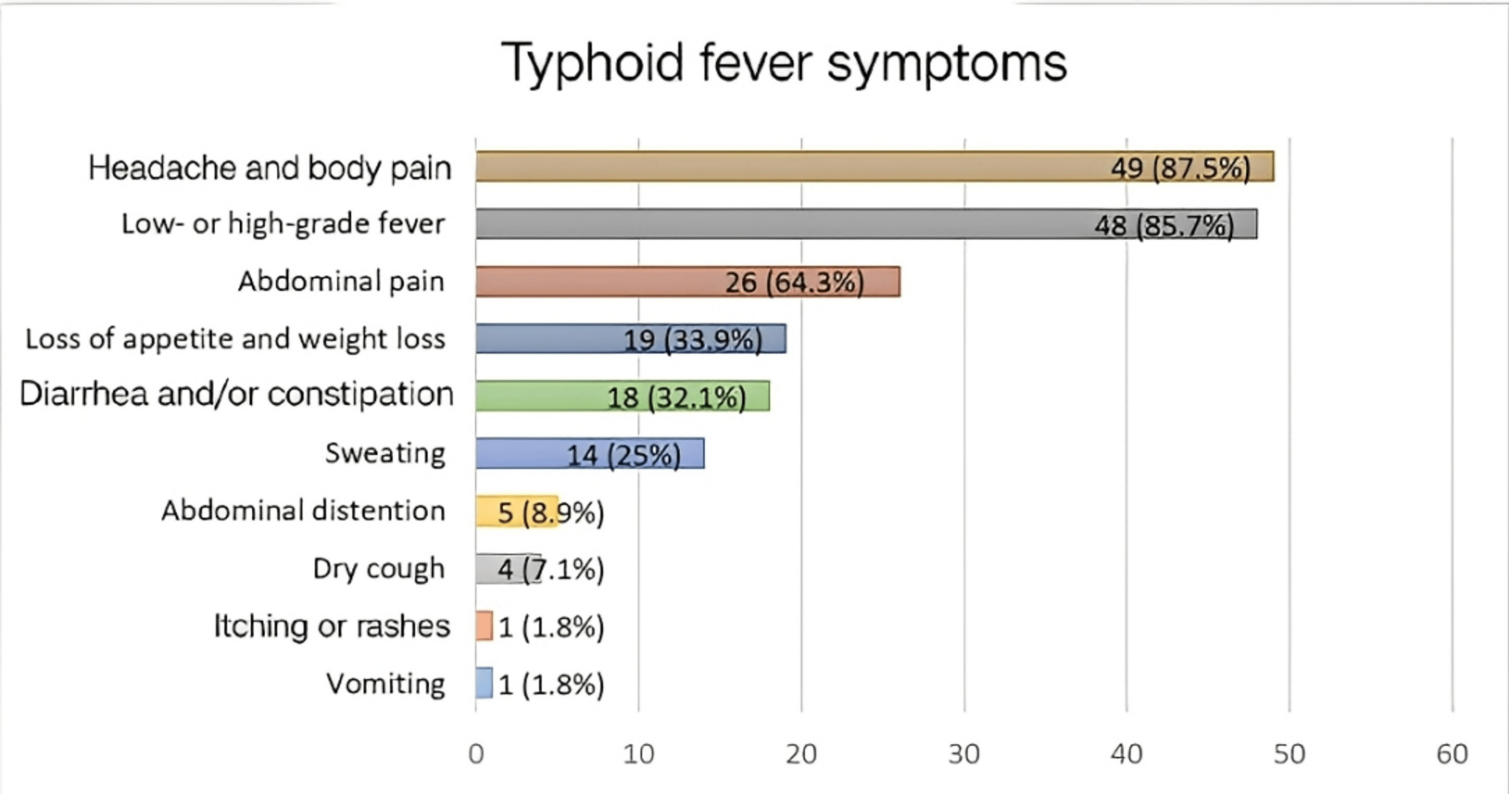
Frequency of typhoid fever symptoms among patients at Kosti Teaching Hospital

A total of 56 questionnaires were collected from patients diagnosed with typhoid fever at Kosti Teaching Hospital. The most frequently reported symptoms were headache and body pain, observed in 49 patients (87.5%), followed by fever in 48 patients (85.7%), abdominal pain in 36 patients (64.3%), and loss of appetite and weight in 19 patients (33.9%). Less common symptoms included dry cough in four patients (7.1%), itching or rashes in one patient (1.8%), and vomiting in one patient (1.8%) (Figure 1).

**Figure 2:**
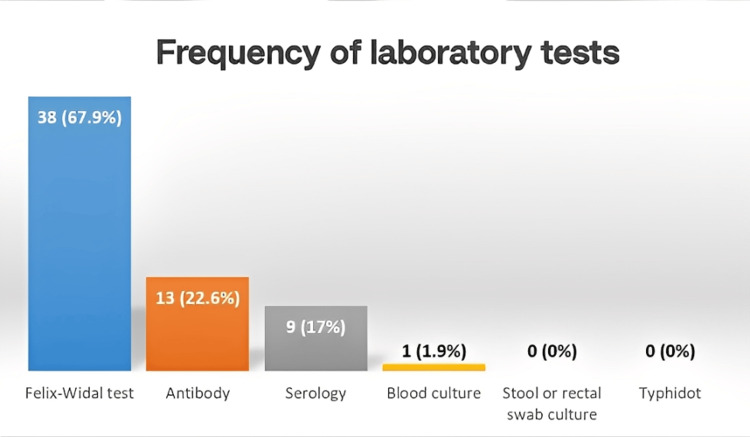
Frequency of laboratory tests performed among participants at Kosti Teaching Hospital

Regarding laboratory tests, the Felix-Widal test was the most frequently used method for diagnosing typhoid fever, performed in 38 patients (67.9%). Detection of serum antibodies was conducted in 13 patients (22.6%), serology in nine patients (17%), and blood culture in one patient (1.9%). Stool or rectal swab culture and the Typhidot^®^ test were not performed for any of the participants (Figure 2).

## Discussion

This clinical audit highlights the diagnostic practices for enteric fever at Kosti Teaching Hospital and underscores the considerable gaps between current practices and internationally recommended standards. Enteric fever remains a significant public health problem in Sudan and other LMICs, primarily due to poor sanitation, limited access to safe water, and fragile healthcare infrastructure [1,2]. The audit findings revealed a heavy reliance on the Widal test (67.9%) and a striking underutilization of blood culture (1.9%), the gold-standard diagnostic method. These results are consistent with previous studies in Sudan and similar endemic regions, where most diagnoses are based on serological tests or purely clinical suspicion [7,10,27].

The Widal test, although widely used, has long been criticized for its poor sensitivity and specificity, particularly in endemic areas where background antibody levels are high [26]. This leads to frequent false positives and contributes to the overdiagnosis of typhoid fever [11]. In Kosti, as in many parts of Sudan, resource constraints, lack of trained laboratory personnel, and low availability of blood culture facilities explain the dependence on the Widal test. However, overreliance on this test risks inappropriate antibiotic use and mismanagement of febrile illnesses, especially since malaria, brucellosis, and viral infections may mimic typhoid clinically [27,29].

Blood culture remains the diagnostic gold standard, with specificity approaching 100% [23]. Nevertheless, its sensitivity is limited to 40-60%, particularly if patients receive antibiotics before sample collection [25]. Despite these challenges, expanding access to blood culture would improve diagnostic accuracy, enable antimicrobial susceptibility testing, and guide evidence-based treatment [25,28]. Unfortunately, our audit found that this tool was rarely employed, reflecting systemic challenges in laboratory infrastructure. Bone marrow cultures, while more sensitive, are invasive and impractical for routine use in Sudan [24].

RDTs and Typhidot assays, which detect specific IgM and IgG antibodies, were not used in the audit population. Although some studies have reported moderate sensitivity and specificity for these tests, their performance varies widely, and none have yet replaced blood culture as a reliable standard [26]. Nevertheless, introducing validated RDTs could complement culture-based diagnosis in resource-limited settings.

The findings also revealed that clinical features such as fever, abdominal pain, and constitutional symptoms remained the predominant diagnostic criteria. This reflects a global trend in LMICs, where up to 60% of typhoid diagnoses are made on clinical grounds alone [28]. While syndromic diagnosis is practical, it risks both over- and under-diagnosis. Overdiagnosis, in particular, fuels unnecessary antibiotic prescriptions, as observed in multiple LMIC settings [29]. Such practices contribute to the growing problem of MDR *S. Typhi*, which has been reported in Sudan and across Africa [28].

Comparing these findings to audits in similar contexts, structured interventions can markedly improve outcomes. For example, audits in South Asia demonstrated that implementing SOPs, strengthening laboratory capacity, and training clinicians on diagnostic algorithms reduced inappropriate use of Widal and increased reliance on evidence-based methods [31]. In Sudan, such measures would be highly beneficial, especially when combined with enhanced surveillance and reporting systems.

Limitations

One major limitation of this audit is the reliance on retrospective data, which may have been incomplete or inconsistently recorded. The small sample size and the limited number of blood cultures also constrain generalizability. Selection bias is possible, as data were collected from patients already labeled as having enteric or typhoid fever, rather than from all suspected cases. The exclusion of antibiotic prescription practices due to inappropriate documentation is another limitation. Additionally, demographic data of the patients were not included, as the audit focused solely on diagnostic methods. Nonetheless, the audit provides critical insights into prevailing diagnostic practices and highlights urgent areas for intervention.

## Conclusions

This audit identified major gaps in the diagnostic practices for enteric fever at Kosti Teaching Hospital, where reliance on clinical assessment and Widal testing predominates, while WHO-recommended blood culture is rarely used due to limited resources and laboratory capacity. Such practices undermine diagnostic accuracy and contribute to unreliable diagnoses. The findings highlight systemic challenges related to laboratory capacity, availability of diagnostic tools, and clinician reliance on low-specificity tests.

This audit also underscores broader health system concerns. The scarcity of diagnostic tools reflects systemic underfunding, while dependence on inaccurate tests perpetuates poor patient outcomes. Addressing these issues requires strengthening laboratory infrastructure, ensuring access to reliable diagnostic tools, and providing continuous training for healthcare workers. Enhancing these areas will align diagnostic practices with WHO standards, improve patient outcomes, and support national efforts to control enteric fever in Sudan.

## Appendices

Questionnaire

Diagnosis of typhoid fever

If you or someone you know is exhibiting symptoms of typhoid, seek medical attention immediately.

A. The symptoms include:

1. Low to high fever

2. Headache and body pain

3. Loss of appetite and weight Loss

4. Dry cough

5. Sweating

6. Abdominal pain

7. Swelling in the abdomen

8. Diarrhea or constipation

9. Itching or rashes

B. Lab

1. Blood cultured

2. Serum for serological

3. Stool or rectal swab culture

4. Felix-Widal test

5. Specific IgM and IgG antibodies

6. Typhidot^®^ test
